# Identification of Plastoglobules as a Site of Carotenoid Cleavage

**DOI:** 10.3389/fpls.2016.01855

**Published:** 2016-12-08

**Authors:** Sarah Rottet, Julie Devillers, Gaétan Glauser, Véronique Douet, Céline Besagni, Felix Kessler

**Affiliations:** ^1^Laboratory of Plant Physiology, Institute of Biology, University of NeuchâtelNeuchâtel, Switzerland; ^2^Neuchâtel Platform of Analytical Chemistry, University of NeuchâtelNeuchâtel, Switzerland

**Keywords:** plastoglobule, *ccd4*, carotenoid, senescence, chloroplast, *Arabidopsis thaliana*

## Abstract

Carotenoids play an essential role in light harvesting and protection from excess light. During chloroplast senescence carotenoids are released from their binding proteins and are eventually metabolized. Carotenoid cleavage dioxygenase 4 (CCD4) is involved in carotenoid breakdown in senescing leaf and desiccating seed, and is part of the proteome of plastoglobules (PG), which are thylakoid-associated lipid droplets. Here, we demonstrate that CCD4 is functionally active in PG. Leaves of *Arabidopsis thaliana ccd4* mutants constitutively expressing CCD4 fused to yellow fluorescent protein showed strong fluorescence in PG and reduced carotenoid levels upon dark-induced senescence. Lipidome-wide analysis indicated that β-carotene, lutein, and violaxanthin were the principle substrates of CCD4 *in vivo* and were cleaved in senescing chloroplasts. Moreover, carotenoids were shown to accumulate in PG of *ccd4* mutant plants during senescence, indicating translocation of carotenoids to PG prior to degradation.

## Introduction

Plastoglobules (PG) are chloroplast lipoprotein particles surrounded by a lipid monolayer and associated with the thylakoid membrane via the outer lipid leaflet. PG were first thought only to be lipid storage sites. But a series of studies revealed that PG actively participate in lipid synthesis and repair (Eugeni Piller et al., 2012; Rottet et al., 2015; Spicher and Kessler, 2015). It was shown that PG contain enzymes such as tocopherol cyclase (VTE1), NAD(P)H dehydrogenase C1 (NDC1), and phytyl ester synthase (PES), as well as others (Zbierzak et al., 2010; Eugeni Piller et al., 2011, 2014; Lippold et al., 2012). Subsequently, roles for PG in synthesis and metabolism of plastochromanol, tocopherol, phylloquinone, fatty acid phytyl esters, and triacylglycerol were demonstrated. In chromoplasts, PG accumulate large amounts of carotenoid esters and are implicated in carotenoid biosynthesis. Carotenoid biosynthesis enzymes are recruited to chromoplast PG presumably to channel intermediates and streamline carotenoid production (Ytterberg et al., 2006). A homolog of PES1, pale yellow petal 1 (PYP1) has been implicated in the formation of the abundant carotenoid esters in chromoplasts (Ariizumi et al., 2014). In chloroplasts, carotenoid biosynthetic enzymes associate with membranes rather than PG (Ruiz-Sola and Rodríguez-Concepción, 2012). Little is known about the catabolic fate of photosynthesis-related carotenoids in senescent chloroplasts (Tevini and Steinmüller, 1985; Biswal, 1995). But, it has been shown that carotenoid cleavage dioxygenase 4 (CCD4) is present in the PG proteome (Vidi et al., 2006; Ytterberg et al., 2006; Lundquist et al., 2012) suggesting a role of PG in carotenoid cleavage.

CCD4 belongs to the carotenoid cleavage dioxygenase family, which has nine members in *Arabidopsis thaliana*. Across the plant kingdom, CCD4 was shown to be a multifunctional enzyme that carries out a variety of closely related cleavage reactions depending on the developmental stage and tissue (Ohmiya et al., 2006; Rubio et al., 2008; Huang et al., 2009; Ahrazem et al., 2010; Campbell et al., 2010; Rodrigo et al., 2013; Ma et al., 2014; Zhang et al., 2015). In *A. thaliana* (At) the exact reaction mechanisms remain unsolved. First insight into the biological function of AtCCD4 arose from a PG proteome study, where dark treatment resulted in a twofold accumulation of CCD4 in PG compared to high light treatment (Ytterberg et al., 2006). Therefore, AtCCD4 was predicted to play a role in dark-induced breakdown of carotenoids. Later, it was observed that AtCCD4 was downregulated in the *abc1k1 abc1k3* kinase double mutant, which may explain the increased level of carotenoids measured in PG of *abc1k1 abc1k3* when compared to wild type (WT) (Lundquist et al., 2013). Using linkage mapping and genome-wide association studies, *AtCCD4* was identified as a negative regulator of carotenoid content during seed desiccation (Gonzalez-Jorge et al., 2013). The same study also showed that it was implicated in carotenoid breakdown during dark-induced senescence in leaves. In both seed and leaf, β-carotene was the most affected among a selection of carotenoids (Gonzalez-Jorge et al., 2013). More recently, *AtCCD4* was implicated in the formation of different apocarotenoids that serve as signaling molecules (Avendano-Vazquez et al., 2014; Lätari et al., 2015).

This article presents data regarding AtCCD4 localization and function. Co-expression with a fluorescent PG marker protein as well as membrane fractionation, provided strong evidence for AtCCD4 localization in PG. Senescence was induced before lipidome-wide analysis to further characterize the biochemical phenotype of the *ccd4* mutants and CCD4 complemented lines. Importantly, the study also demonstrates accumulation of carotenoid substrates in PG of the *ccd4* mutant under natural senescence. In summary, the data indicate that PG are a site of carotenoid cleavage adding another function to this chloroplast subcompartment.

## Results

### Subcellular Localization of AtCCD4 in Plastoglobules

**FIGURE 1:**
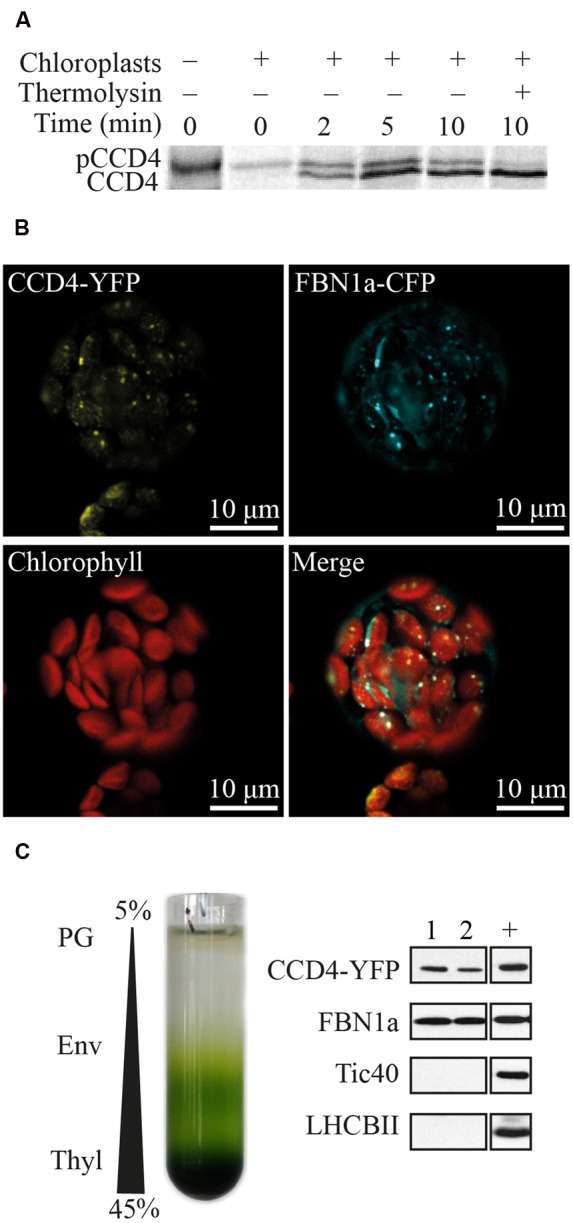
**Plastoglobule localization of CCD4. (A)** Pre-[^35^S] CCD4-6xHIS (pCCD4) was incubated with isolated Pea chloroplasts from 0 to 10 min *in vitro*. Upon import, mature [^35^S] CCD4-6xHIS (CCD4) showed a lower molecular mass due to the removal of the transit peptide and unlike pCCD4 was resistant to thermolysin protease. **(B)** Colocalization of CCD4-YFP and FBN1a-CFP in isolated protoplasts of *Arabidopsis thaliana*. **(C)** Isolation of PG by chloroplast membrane fractionation on a discontinuous sucrose gradient (PG, plastoglobules; Env, envelopes; Thyl, thylakoids). Pure PG fractions (nos. 1 and 2) contain recombinant CCD4-YFP. Tic40, FBN1a and LHCBII are envelope, PG and thylakoid markers, respectively. +, positive control which corresponds to a membrane fraction containing all four markers.

*CCD4* (At4g19170) encodes a protein of 595 amino acids with a predicted molecular weight of 65.6 kDa. A N-terminal chloroplast transit peptide of 34 residues was predicted by ChloroP algorithm^1^. Targeting of CCD4 to the chloroplast and the processing of the predicted transit peptide was assessed in an *in vitro* import assay using isolated pea chloroplasts (**Figure 1A**). As demonstrated, pre-[^35^S] CCD4-6xHIS was imported and processed inside the chloroplasts to a mature thermolysin-resistant protein of the expected mass.

Subcellular localization of CCD4 was analyzed by confocal microscopy of protoplasts prepared from *A. thaliana ccd4-2* mutants complemented with CCD4-YFP (yellow fluorescent protein), which were subsequently transformed with a construct encoding FBN1a-CFP (cyan fluorescent protein) (**Figure 1B**). Chloroplasts were identified by their red chlorophyll autofluorescence. CCD4-YFP, as well as the PG marker FBN1a-CFP (Vidi et al., 2006), resulted in punctate fluorescence inside the chloroplasts. Moreover, the two colocalized in the fluorescent spots, most likely PG.

To exclude that the fluorescent spots for CCD4-YFP were non-specific protein aggregates, PG were isolated from transgenic plants by sucrose gradient floatation (**Figure 1C**). Due to their low density, PG floated to the top of the gradient and were easily isolated by recovering the two first fractions. To evaluate the purity of the PG fractions, the following proteins were used as membrane markers: light-harvesting chlorophyll *a*/*b*-binding 2 (LHCBII) for thylakoid membranes, translocon at the inner envelope membrane of chloroplasts 40 (Tic40) for the chloroplast envelope membranes, and FBN1a (also known as PGL35) a well-accepted marker for PG (Lundquist et al., 2012). Pure PG, as supported by the presence of FBN1a and the absence of Tic40 and LHCBII, contained the CCD4-YFP fusion protein (detected by an HA-tag included in the YFP moiety).

### Plastoglobules of Senescent *ccd4* Contain More β-Carotene and Lutein

**FIGURE 2:**
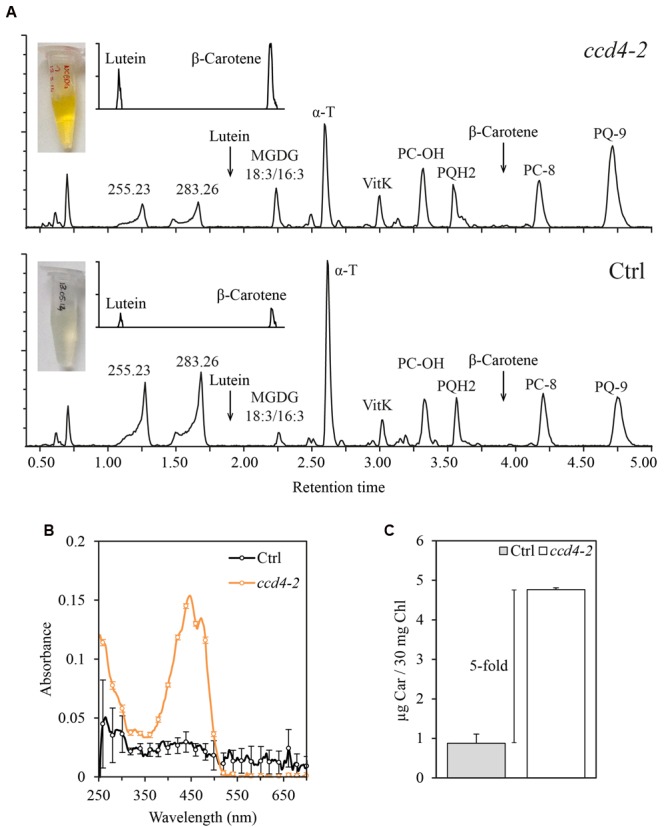
**Carotenoids accumulated in senescent plastoglobules of *ccd4-2*. (A)** PG were isolated from naturally senescent *ccd4-2* and control plants (3 months old) by a sucrose gradient loaded with a chlorophyll-equivalent of 30 mg of membranes. Lipids from the PG fractions were analyzed by untargeted ultra-high pressure liquid chromatography-atmospheric pressure chemical ionization-quadrupole time of flight mass spectrometry and chromatograms of detected lipids are shown. **(B)** Spectrophotometric analysis of PG fractions. Three maxima around 450 nm, typical of carotenoid absorption spectra, were detected in *ccd4-2* PG. **(C)** Quantification of total carotenoid contents in PG fractions. Absorbance at 448 nm was recorded and compared with a calibration curve of β-carotene standard. Carotenoid content is expressed in μg of β-carotene per 30 mg of chlorophyll of total membranes used in the experiment. MGDG, monogalactosyldiacylglycerol; α-T, α-tocopherol; VitK, vitamin K; PC-OH, hydroxy-plastochromanol; PQH2, plastoquinol; PC-8, plastochromanol; PQ-9, plastoquinone; Ctrl, control; Car, β-carotene; Chl, chlorophyll.

Because of the localization of CCD4 at PG and the known role of PG in senescence (Besagni and Kessler, 2013; Springer et al., 2015), we investigated the lipid composition of PG during natural senescence in *ccd4-2*. As a control, the experiment was simultaneously carried out on an unrelated senescing transgenic line. PG were purified by floatation on a discontinuous sucrose gradient using isolated chloroplast membranes (corresponding to 30 mg of chlorophyll). The visible difference between the two PG fractions was striking. The PG fraction from the *ccd4-2* mutant appeared orange, while it was normally pale-yellow as seen in the PG fraction from the control line (**Figure 2A**).

To investigate the composition of *ccd4-2* PG, lipid profiling was carried out. Major compounds detected by this method are shown by comparably scaled chromatograms in **Figure 2A**. The typical PG prenylquinones were observed in both samples, namely α-tocopherol, vitamin K, plastochromanol and plastoquinone. Monogalactosyldiacylglycerol (MGDG) 18:3/16:3 appeared as the dominant galactolipid in both PG extracts. Two compounds at m/z 255.2320 (RT: 1.25 min) and m/z 283.2632 (RT: 1.66 min) were tentatively identified as fatty acids 16:0 (C_16_H_31_O_2_, error = -1.6 ppm) and 18:0 (C_18_H_35_O_2_, error = -1.8 ppm), respectively. To visualize carotenoids such as β-carotene and lutein, a magnification of the corresponding chromatogram is shown in the upper left corner.

In order to compare lipid levels in PG, the normalization of PG concentration is critical. We thus considered the total amount of prenylquinones as an internal standard for PG concentration (Lundquist et al., 2013). Relative intensities of α-tocopherol-quinone, α-, δ-, γ-tocopherol, vitamin K, plastochromanol-8 and plastoquinone were added up and used to normalize data. Noteworthy, these prenylquinone contents were not significantly affected by the loss of CCD4 in a whole plant extract. Variation between *ccd4-2* and control was obtained by calculating the fold change of lipid normalized intensity. Results are summarized in **Table 1** and showed a fivefold and a threefold increase in concentration of β-carotene and lutein, respectively, in *ccd4-2*. In addition, galactolipids MGDG 18:3/16:3 and 18:3/18:3 were about two to three times more abundant in PG of *ccd4-2*.

**Table 1:** Lipid content of senescent *ccd4-2* PG compared to control.

PG lipid	Fold change
β-Carotene	5.2
MGDG-18:3/16:3	3.3
Lutein	3.2
MGDG-18:3/18:3	2.1
Plastoquinone	2.0
Vitamin K	1.4
Plastoquinol	1.1
Plastochromanol	1.1
α-Tocopherol-quinone	-1.0
DGDG-18:1/18:3	-1.2
MGDG-18:2/18:3	-1.4
α-Tocopherol	-1.5
δ-Tocopherol	-1.6
γ-Tocopherol	-1.8

*Relative peak intensity from chromatograms shown in **Figure 2A** was normalized for total prenylquinones prior to calculating the fold change between *ccd4-2* and control.*

In addition, a spectroscopic analysis was carried out on isolated PG shown in **Figure 2A** to evaluate the overall carotenoid content by an independent method. The absorption spectra of the PG lipid extracts revealed carotenoid-typical peaks with maxima at 420, 450, and 470 nm for *ccd4-2* which were absent in the control (**Figure 2B**). Absolute quantification using a commercial β-carotene standard showed a fivefold higher carotenoid content in *ccd4-2* PG compared to that in the control (**Figure 2C**).

### Characterization of CCD4 Complemented Plants

**FIGURE 3:**
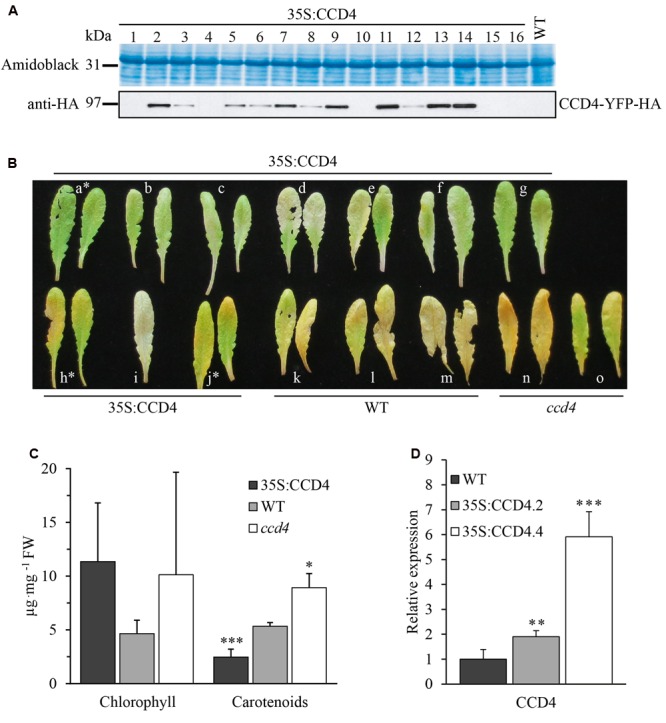
**Complementation of *ccd4* mutants with 35S:CCD4-YFP-HA. (A)** Relative amounts of CCD4-YFP-HA in complemented lines were assessed by immunoblot analysis with anti-HA antibodies. A representative blot of 16 independent lines is shown, none of which showed a visible phenotype. **(B)** Complementation analysis of CCD4 complemented lines by dark-induced senescence. Detached leaves were subjected to dark-induced senescence for 10 days and complementation was determined based on the phenotype. Two 35S:CCD4 plants (h and j) were not complemented as they showed a yellowish color similar to *ccd4-4.* **(C)** Total chlorophyll and carotenoids were extracted from the leaves shown in **(B)** (NB: the white asterisks mark the leaves that were excluded for the calculations). Concentrations of pigments were established with a spectrophotometer. Means ± SD were calculated for CCD4 complemented plants (*n* = 7), WT *(n* = 3) and *ccd4 (n* = 2). Statistical significance was determined by a Student’s *t*-test (unpaired homoscedastic, two-tailed, ^∗^*P* < 0.05, ^∗∗∗^*P* < 0.001). **(D)** CCD4 gene expression analysis. Transcripts of CCD4 were quantified by qPCR in the WT and in the two complemented lines, i.e., 35S:CCD4.2 and 35S:CCD4.4. The relative expression of CCD4 was normalized to the reference gene *ACTIN*. Means ± SD (*n* = 6). Statistical significance between transgenic lines and the WT was determined by a Student’s *t*-test (unpaired heteroscedastic, two-tailed, ^∗∗^*P* < 0.01, ^∗∗∗^*P* < 0.001).

To investigate CCD4 subcellular localization (shown in **Figure 1**) and *in vivo* function, *A. thaliana ccd4-2* and *ccd4-4* mutants (Scholl et al., 2000; Gonzalez-Jorge et al., 2013) were transformed with a construct carrying a glufosinate resistance marker and a fusion between CCD4 and YFP tagged with human influenza hemagglutinin (HA) under control of the constitutive cauliflower mosaic virus 35S promoter. Resulting CCD4 complemented plants were *ccd4-2*::35S:CCD4-YFP (abbreviated as 35S:CCD4.2) and *ccd4-4*::35S:CCD4-YFP (abbreviated as 35S:CCD4.4). Ten independent glufosinate-resistant T1 plants were selected based on a visible band at the expected mass of 94.7 kDa by immunoblotting with anti-HA (**Figure 3A**). T2 progeny of nine lines segregated 3:1 (glufosinate resistant to glufosinate sensitive) indicating a single insertion of the transgene. Among T3 progeny, six independent plants were identified by segregation analysis as being homozygous for the insertion of the *35S:CCD4-YFP* construct. CCD4 complemented plants showed a WT phenotype regardless of the *ccd4* mutant background. To compare CCD4-YFP expression in transgenic plants with that of CCD4 in WT plants we carried out qPCR reactions. The relative expression of 35S:CCD4.2 and 35S:CCD4.4 were around two and six-times higher than WT (**Figure 3D**).

Complementation was assessed by phenotypic observation under dark-induced senescence in leaves. Surprisingly, most of the CCD4 complemented lines developed a less yellowish leaf phenotype than WT, suggesting that their carotenoid content was lower. Some lines (e.g., h and j) also showed yellowish leaves similar to the corresponding *ccd4-4* mutant (**Figure 3B**). However, in most transgenic lines CCD4-YFP appeared to complement *ccd4*, which is evident from the lack of yellowing of the leaves upon senescence. Note that chlorophyll distribution seemed irregularly affected in both *ccd4* and CCD4 complemented lines (**Figure 3B**).

To quantify the visual observations, total chlorophyll and carotenoids were rapidly extracted and analyzed by spectrophotometry (**Figure 3C**). As expected, the CCD4 complemented lines contained significantly (*P* < 0.001) less carotenoids than the WT. In contrast, *ccd4* contained significantly (*P* < 0.05) more carotenoids than the WT. Chlorophyll contents showed no statistical difference among the genotypes. In conclusion, the constitutive expression of *CCD4-YFP* did not cause an apparent phenotype under standard growth conditions. However, the complemented plants exhibited elevated carotenoid degradation under dark-induced senescence, as supported by less yellowish leaves and reduced total carotenoid contents.

### Enhanced Carotenoid Degradation in CCD4 Complemented Plants

**FIGURE 4:**
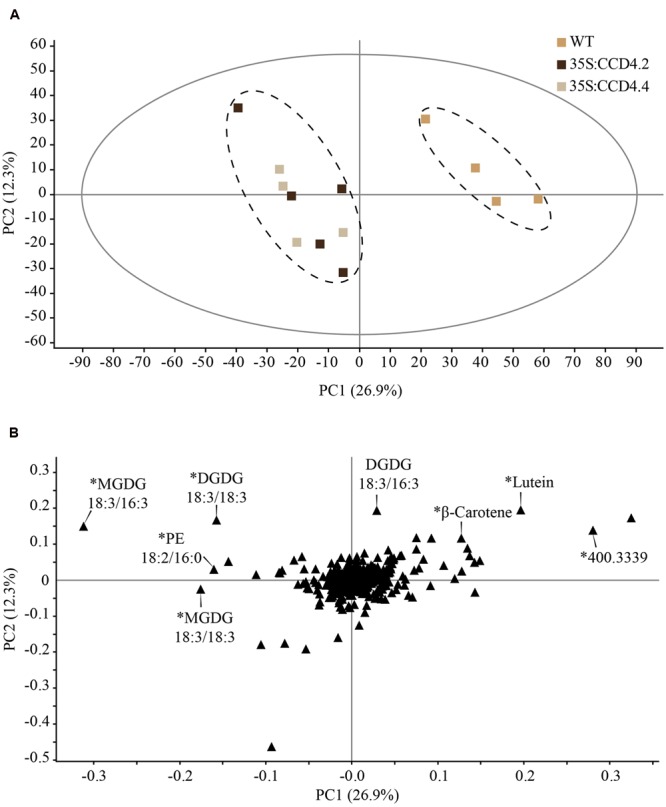
**Constitutive expression of CCD4 resulted in a distinct lipid profile during dark-induced senescence in leaves when compared with WT. (A)** Detached leaves were placed in darkness for 8 days prior to lipid extraction (*n = 5*). Lipids were analyzed by ultra-high pressure liquid chromatography-atmospheric pressure chemical ionization-quadrupole time of flight mass spectrometry. Discrimination was evaluated with a partial least squares-discriminant analysis in which outlying samples were removed prior to data processing. **(B)** Compounds responsible for the difference between CCD4 complemented lines and WT are indicated on the loading plot. ^∗^Statistically significant according to Student’s *t*-test (unpaired heteroscedastic, two-tailed). PC, principal component.

To assess the effects of constitutive *CCD4-YFP* expression on the degradation of carotenoids, we investigated the lipid contents in leaves before and after dark-induced senescence of CCD4 complemented *A. thaliana* plants. Data obtained from untargeted ultra-high pressure liquid chromatography-atmospheric pressure chemical ionization-quadrupole time of flight mass spectrometry (UHPLC-APCI-QTOFMS) was subjected to partial least squares-discriminant analysis (PLS-DA) in order to investigate significant differences between treatments. Our findings underlined an absence of statistical difference between WT and CCD4 complemented plants under standard growth conditions, whereas senescence resulted in genotype-clustering with the first component explaining 26.9% of the variability (**Figure 4A**). Under senescence conditions, the PLS-DA model was validated with a permutation test (*n =* 200; *Q*_2_ = 0.0, -0.218). As expected, 35S:CCD4.2 and 35S:CCD4.4 displayed a similar lipid profile, which was different from that of WT. The loading plot was investigated to point out the main compounds responsible for the discrimination between WT and CCD4 complemented lines. Among more than 300 lipids measured, the few outlying compounds were identified whenever possible (**Figure 4B**) and relative quantifications subjected to Student’s *t*-test. In brief, WT accumulated significantly more lutein and β-carotene than CCD4 complemented plants. What is more, an unidentified compound at m/z 400.3339 (RT: 2.41 min, C_27_H_44_O_2_) was three times higher in WT. Polar lipids such as MGDG 18:3/16:3, 18:3/18:3, DGDG 18:3/18:3, and PE 18:2/16:0 were more abundant in CCD4 complemented plants.

**FIGURE 5:**
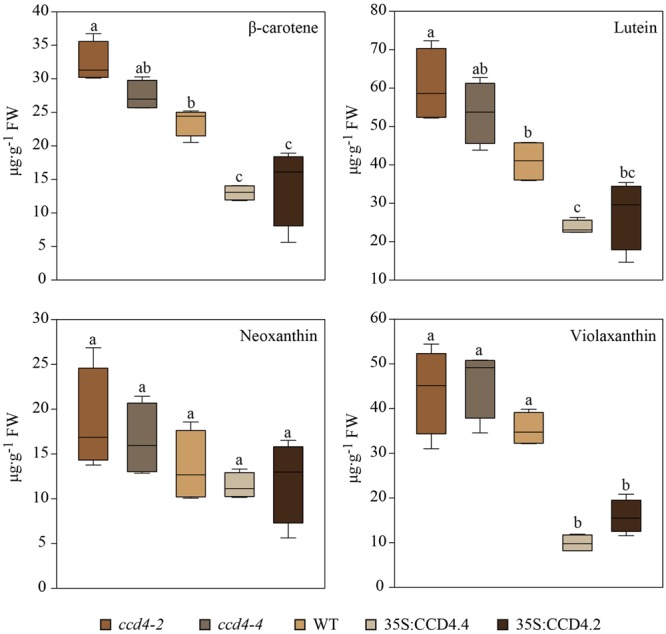
**Reduced level of photosynthesis-related carotenoids during senescence in CCD4 complemented plants.** Detached leaves were placed in darkness for 8 days prior to lipid extraction (*n = 5*). Lipids were separated by UHPLC-DAD following the carotenoid-profiling protocol. Absolute quantification of carotenoids was carried out on the basis of calibration curves obtained with pure standards. Box plots created with SigmaPlot are shown. Statistical differences across genotypes were assessed with a one-way ANOVA. Pairwise multiple comparisons (Holm–Sidak method) revealed significant differences (letters are distributed accordingly, *P* < 0.05).

The carotenoid-targeted UHPLC analysis enabled an absolute and accurate quantification of carotenoids (**Figure 5**). On the whole, total carotenoid levels in senescent leaves of CCD4 complemented plants (63.7 μg g^-1^ FW (fresh weight)) were 1.8 times lower than those of the WT (113.57 μg g^-1^ FW) and 2.3 times lower than those of *ccd4* (149.4 μg g^-1^ FW). The contents of β-carotene, lutein and violaxanthin were all significantly reduced in CCD4 complemented plants while neoxanthin was not. Neoxanthin and violaxanthin contents were the least affected in the *ccd4* mutant. In contrast, violaxanthin was reduced about threefold in CCD4 complemented plants compared with WT.

## Discussion

CCD4 has a predicted cleavable N-terminal chloroplast transit peptide. In agreement with bioinformatics predictions and a previous study (Naested et al., 2004), CCD4 was successfully imported into isolated chloroplasts demonstrating chloroplast localization (**Figure 1**). The association of CCD4 with PG was addressed using two independent approaches: (a) colocalization with a known PG marker and (b) subfractionation of chloroplasts. Both approaches provided clear evidence for association of CCD4 with PG (**Figure 1**). The association of CCD4 with PG provides the molecular framework for a role of this chloroplast subcompartment in carotenoid cleavage.

The localization of CCD4 in PG suggests that during senescence carotenoids move to PG to be cleaved. Therefore accumulation of uncleaved carotenoids in *ccd4* mutant PG may be expected. We used a lipidomics approach to address the role of CCD4 and PG in carotenoid breakdown. For this purpose, PG from *ccd4* mutant and control plants were analyzed. Even visually, *ccd4* PG fractions were distinctly more orange than the control (**Figure 2A**). This observation was confirmed by untargeted lipid analysis demonstrating that they contained fivefold and threefold more β-carotene and lutein, respectively (**Table 1**). ABC1K1 and -K3 are regulatory kinases associating with PG (Lundquist et al., 2013; Martinis et al., 2013, 2014). In the corresponding double mutant, the *CCD4* gene was downregulated and PG contained higher levels of carotenoids than the WT (Lundquist et al., 2013). These earlier findings provide additional evidence for the role of CCD4 and PG in carotenoid cleavage.

Our observations are in agreement to Gonzalez-Jorge et al. (2013), which reported CCD4 as being a major negative regulator of β-carotene in senescent leaves. Here, constitutive overexpression of CCD4 in the *ccd4* mutant background increased degradation of carotenoids upon senescence (**Figure 3**) but under standard growth conditions the carotenoid content was indistinguishable from the WT. This observation may be explained in the following way: in vegetative chloroplasts, carotenoids associate with the light-harvesting complex (LHC) proteins in the thylakoids (Croce et al., 1999; Li et al., 2000; Dall’Osto et al., 2010). Under these conditions carotenoids are stably bound and inaccessible to CCD4. Only upon senescence would LHC be proteolytically degraded. The carotenoids are then free to diffuse to PG for CCD4-dependent cleavage.

Interestingly, the untargeted lipid analysis of senescing overexpressing CCD4 plants also indicated higher levels of galacto- and phospholipids than in the WT (**Figure 4**). These lipids are major constituents of the thylakoid membrane. It is therefore possible that the degradation of thylakoid membranes in CCD4 overexpressing plants is retarded under senescence conditions. Potentially, CCD4-dependent carotenoid cleavage products, commonly termed apocarotenoids, may serve as signal ensuring the synchronization of membrane breakdown-related processes (Auldridge et al., 2006; Walter et al., 2010). For instance, *AtCCD4* was implicated in the formation of an unknown apocarotenoid that serves as a signaling molecule in *zeta-carotene desaturase* (*zds*), an albino mutant arrested in early chloroplast development and defective in carotenoid synthesis (unable to convert ζ-carotene into lycopene) (Avendano-Vazquez et al., 2014). Moreover, *AtCCD4* was correlated with the formation of C_13_-apocarotenoid glycosides that occur in overexpressors of phytoene synthase (PSY), which catalyzes the rate-limiting step in carotenoid biosynthesis (Lätari et al., 2015). Taken together, these results suggest that CCD4 acts on carotenoid turnover in leaves of *A. thaliana* producing apocarotenoids that may be metabolized further. Through this activity, a role of CCD4 in the production of retrograde signaling molecules that relay information on the chloroplast state for downstream gene regulation appears likely.

Untargeted as well as targeted analyses identified lutein, β-carotene and violaxanthin as the most important targets of CCD4 during senescence (**Figures 4** and **5**). When comparing all genotypes, the carotenoids that accumulated in *ccd4* were cleaved more extensively in CCD4 overexpressing plants than in the WT providing additional evidence for the role of CCD4 (**Figure 5**). However, our analysis did not identify a distinct carotenoid cleavage product. This suggests that cleavage may result in a variety of products including volatiles that may escape identification by liquid chromatography-mass spectrometry. The data in this paper indicate PG as a site of carotenoid-cleavage in senescent chloroplasts. In the future it will be of interest to identify the cleavage products and their potential functions as signaling molecules.

## Materials and Methods

### Plant Material and Growth Conditions

*Arabidopsis* WT was Columbia-0. Two T-DNA insertion mutants from *A. thaliana, ccd4-2* (Salk_097984) and *ccd4-4* (Salk_010751), disrupted in *CCD4* (At4g19170) gene were obtained from the European *Arabidopsis* Stock Centre (Scholl et al., 2000). Seeds were surface sterilized (0.05% v/v Triton X-100, 70% v/v ethanol) and sowed on solidified ½ 12 MS (Murashige and Skoog) medium (Murashige and Skoog, 1962). Germination was induced and synchronized at 4°C for 2 days. Plants were grown under short day conditions (8 h light, 16 h dark, 150 μmol m^-2^ s ^-1^) in a GroBank system (CLF Plant Climatics). Afterward, plants were further grown on soil (Ricoter). Alternatively, seeds were directly sowed on soil.

Senescence was induced by detaching the whole rosette from roots and placing it on a filter paper slightly humidified (tap water) in a Petri dish for 7 days in the dark. Alternatively, leaves were detached from the whole rosette and subjected to dark treatment as per described.

### Plasmid Construction

Genomic DNA from *A. thaliana* was used to amplify *CCD4* gene by PCR using the primer couple CCD4TopoF (5′-CACCATGGACTCTGTTTCTTCTTCTTCC-3′) and CCD4TopoR (5′-CCATGGAAGCTTATTAAGGTCACTTTCC-3′). The PCR product was inserted into the pENTR D-TOPO entry vector using the Gateway^®^ BP Clonase^®^ II Enzyme Mix (Invitrogen). *CCD4* was then recombined from the entry vector to two other Gateway-based destination vectors (Gateway^®^ LR Clonase^®^ II Enzyme Mix, Invitrogen), namely p0GWA bacterial expression vector (Busso et al., 2005) and pEarleyGate101 plant expression vector (Earley et al., 2006). Respectively, the constructs obtained were *T7:CCD4-6xHIS* and *35S:CCD4-YFP-HA*.

### Complementation of *ccd4* with 35S:CCD4-YFP-HA

The pEarlyGate101-CCD4 construct was introduced by electroporation into the *Agrobacterium tumefaciens* C58 strain. Flowering *A. thaliana* of *ccd4* (*ccd4-2* and *ccd4-4)* was transformed with pEarlyGate101-CCD4 construct using the floral dip method as described (Clough and Bent, 1998). Complemented plants were named 35S:CCD4.2 or 35S:CCD4.4 according to the mutant background in which the transgene was expressed (*ccd4-2* or *ccd4-4*, respectively). Transgenic plants were selected on solidified ½ 12 MS medium supplemented with 30 mg/L of glufosinate ammonium. Segregation analyses were carried out to select lines with a single insertion of the transgene.

### RNA Extraction, cDNA Synthesis, and qPCR

RNA was isolated from 2-week-old plants grown on ½ 12 MS medium (supplemented with 30 mg L^-1^ of glufosinate ammonium for transgenic lines) using the RNeasy Plant Mini Kit from Qiagen, which included a DNase treatment. 1 μg of RNA was used for reverse transcription with the GoScript^TM^ Reverse Transcription System (Promega). qPCR was performed with FastStart Essential DNA Green Master on the LightCycler from Roche. Primers for actin were obtained from Qiagen (Act2 QT00774634) and those for CCD4 were designed as published elsewhere (Gonzalez-Jorge et al., 2013).

### Transformed Protoplasts Observed by Confocal Microscopy

Plants were grown for 3 weeks on ½ 12 MS supplemented with 1% sucrose. For colocalization with CCD4-YFP, the vector pCL62 carrying *FBN1a* in frame with *CFP* was used (Vidi et al., 2006). Protoplasts were isolated and transformed as described (Jin et al., 2001). Fluorescence in protoplasts was monitored 30 h after transformation with a Leica TCS SP5 (Leica Microsystems) confocal microscope using the appropriate parameters for YFP, CFP and chlorophyll autofluorescence. The YFP was excited by the 514-nm Argon laser line with 525–600-nm detection windows. CFP fluorescence was detected using the 458-nm Argon laser line and 465–505-nm detection windows. Settings were checked with untransformed protoplasts for one and both constructs to avoid unspecific background noise.

### Isolation of PG on a Sucrose Gradient

Intact chloroplasts from 8-week-old plants were isolated essentially as described (Hiltbrunner et al., 2001; Kessler and Glauser, 2014). Membrane fractionation, verification by SDS-PAGE and immunoblotting were adapted from published protocols (Vidi et al., 2006; Besagni et al., 2011; Kessler and Glauser, 2014). Lysed and homogenized chloroplasts were subjected to 1 h of ultracentrifugation (Optima XPN-80 Ultracentrifuge, Beckman Coulter) to separate the total membranes from the stroma. Subsequently, homogenized membranes (corresponding to 3.3 mg of chlorophyll) were separated with a miniaturized discontinuous sucrose gradient as follows. On the top of the 2 mL membrane-containing 45% sucrose, 804 μL of 38%, 804 μL of 20%, 539 μL of 15%, and 845 μL of 5% sucrose were layered in a 5 mL Ultra-Clear^TM^ Centrifuge Tube (13 mm × 51 mm, Beckman Coulter). Gradients were centrifuged at 100,000 × *g* for 15 h in a SW55Ti swinging-bucket rotor (Beckman Coulter). Fractions of 200 μL were collected from the top (no. 1) to the bottom of the gradient (no. 25) with a micropipette.

### Pea Chloroplast Isolation and Protein Import

Intact chloroplasts were isolated from 2- to 3-week-old peas (*Pisum sativum*) as described in Smith et al. (2003) with minor modifications. *In vitro* translated CCD4 was produced from the p0GWA-CCD4 construct with the TNT^®^ Quick Coupled Transcription/Translation System (Promega) following the manufacturer’s recommendations. The ^35^S-radiolabeled CCD4-6xHIS was incubated with isolated import competent pea chloroplasts according to the protocol described in Smith et al. (2003).

### Lipid Extraction from PG for Untargeted UHPLC Analysis

The extraction of lipids from isolated PG was carried out as described (Eugeni Piller et al., 2014; Kessler and Glauser, 2014) with some modifications. To remove sucrose, 400 μL of fraction were partitioned two times against ethyl acetate (1:1). The upper phases were collected, combined, and evaporated. Pellets of lipids were dissolved in 100 μL of tetrahydrofuran for UHPLC-APCI-QTOFMS analysis.

### Total Carotenoid Quantification in PG by Spectrophotometry

From a sucrose-gradient loaded with a chlorophyll-equivalent of 30 mg of membranes (Vidi et al., 2006), 30 μL of the PG fraction was mixed with 100 μL of ethyl acetate. After brief centrifugation in a microfuge, 90 μL of supernatant was collected and evaporated. The pellet was resuspended in 10 μL of ethyl acetate and the visible spectrum was measured using a Nanodrop 1000 Spectrophotometer (Thermo Fisher Scientific). A calibration curve was established in ethyl acetate using β-carotene as the standard. The carotenoid content was determined as the absorbance at 448 nm in ethyl acetate and is expressed as micrograms of β-carotene equivalent per total membrane loaded, which is expressed in milligrams of chlorophyll (μg Car/30 mg of chlorophyll).

### Pigment Quantification in Senescent Leaves by Spectrophotometry

Pigments were extracted from leaves using dimethylformamide (DMF). Leaves (50 mg FW) were finely ground in a microcentrifuge tube using a metallic pestle under liquid nitrogen, after which five volumes of DMF and 5–10 glass beads (Assistant, Sontheim, Germany) were added. Samples were homogenized for 3 min at 30 Hz in a tissue lyser (Retsch MM 300, Haan, Germany). Then, tubes were centrifuged 3 min at 16,000 × *g*. Finally, 2 μL of supernatant were analyzed with a Nanodrop 1000 Spectrophotometer (Thermo Fisher Scientific). Absorbance was recorded at the following four wavelengths: 480, 647, 652, and 664 nm. To determine concentrations of total carotenoids, we applied published equations (Wellburn, 1994). Total chlorophyll was estimated by dividing the absorbance measured at 652 nm by the factor 36.

### Carotenoid Extraction for Targeted UHPLC Analysis

To protect carotenoids from oxidation, the following procedures were carried out in semi darkness. Leaves or rosette tissues (100 mg FW) were ground in a cold mortar in presence of liquid nitrogen, and immediately transferred to a microcentrifuge tube. Five volumes of tetrahydrofuran-methanol (50:50, v/v) and 5–10 glass beads (Assistant, Sontheim, Germany) were added. Samples were further homogenized for 3 min at 30 Hz in a tissue lyser (Retsch MM 300, Haan, Germany). Tubes were centrifuged 5 min at 16,000 × *g* (Eppendorf Centrifuge 5415D) and 400 μL of supernatant was transferred to another 1.5 mL microcentrifuge tube. Centrifugation was repeated and 150 μL of supernatant was transferred to an amber glass vial (BGB, Boeckten, Switzerland) for UHPLC analysis. Samples were either directly analyzed or stored at -80°C for up to 1 week.

Carotenoid quantification was achieved using UHPLC-DAD (diode array detector). The separation was carried out on a Waters Acquity BEH C18 column (50 mm × 2.1 mm, 1.7 μm particle size) held at 50°C at a flow rate of 0.7 mL min^-1^. The following gradient program using water as solvent A and acetonitrile as solvent B was employed: 65–80% B for 5 min, 80–100% B for 1 min, holding at 100% B for 3.5 min, reequilibration at 65% B for 1 min. Total analysis time was 10.5 min. A volume of 2.5 μL was injected. For quantification, the UV trace at 450 nm was used. Calibration solutions were prepared as follows: Lutein, violaxanthin, neoxanthin and zeaxanthin were mixed and diluted at 20, 5, 2, and 0.5 μg mL^-1^ in tetrahydrofuran–methanol–water (42.5:42.5:15, v/v). Since β-carotene is highly apolar, it was separately prepared in tetrahydrofuran–water (85:15, v/v) at 20, 5, 2, and 0.5 μg mL^-1^.

### Lipid Extraction from Whole Plant for Untargeted UHPLC Analysis

For untargeted lipid profiling, pure tetrahydrofuran was used for extraction instead of tetrahydrofuran–methanol. Lipid profiling was performed using a UHPLC-APCI-QTOFMS (Waters) as previously described (Martinis et al., 2011; Kessler and Glauser, 2014). Raw data was processed using Markerlynx XS^TM^ (Waters) for automatic peak detection.

## Author Contributions

FK conceived research plans; CB and FK supervised the experiments; SR and JD performed experiments with the technical assistance of VD; GG carried out lipidomics; SR designed the experiments and analyzed the data; and SR wrote the article with contributions from all of the authors.

## Conflict of Interest Statement

The authors declare that the research was conducted in the absence of any commercial or financial relationships that could be construed as a potential conflict of interest.

## Footnotes

1 http://www.cbs.dtu.dk/services/ChloroP
